# Advancing primary care to promote equitable health: implications for China

**DOI:** 10.1186/1475-9276-11-2

**Published:** 2012-01-20

**Authors:** Li-Mei Hung, Sarika Rane, Jenna Tsai, Leiyu Shi

**Affiliations:** 1 Dept. of Hospitality Management, Hungkuang University, No. 34 Chung-Chie Road, Shalu District, Taichung City 43302, Taiwan, R.O.C; 2 Department of Health Policy and Management, Johns Hopkins Bloomberg School of Public Health, Johns Hopkins Primary Care Policy Center, 624 North Broadway, Baltimore, Maryland, USA; 3 Dept. College of General Education, Hungkuang University, No. 34 Chung-Chie Road, Shalu District, Taichung City 43302, Taiwan, R.O.C; 4 Department of Health Policy and Management, Johns Hopkins Bloomberg School of Public Health, 624 North Broadway, Baltimore, Maryland, USA

## Abstract

China is a country with vast regional differences and uneven economic development, which have led to widening gaps between the rich and poor in terms of access to healthcare, quality of care, and health outcomes. China's healthcare reform efforts must be tailored to the needs and resources of each region and community. Building and strengthening primary care within the Chinese health care system is one way to effectively address health challenges. This paper begins by outlining the concept of primary care, including key definitions and measurements. Next, results from a number of studies will demonstrate that primary care characteristics are associated with savings in medical costs, improvements in health outcomes and reductions in health disparities. This paper concludes with recommendations for China on successfully incorporating a primary care model into its national health policy, including bolstering the primary care workforce, addressing medical financing structures, recognizing the importance of evidence-based medicine, and looking to case studies from countries that have successfully implemented health reform.

## Advancing Primary Care to Promote Equitable Health: Implications for China

Reforming healthcare is a key challenge for almost every country worldwide because there are aspects of each nation's healthcare delivery system that can be improved upon. The fundamental goal of healthcare reform is to promote the equity of health systems; equity includes the following five aspects: (1) broad access to care, (2) expansive coverage, (3) affordable costs for consumers and providers, (4) positive health outcomes, and (5) few disparities [1]. Although it is impossible to eliminate all disparities in healthcare, existing gaps can be narrowed through reform efforts. Healthcare reform priorities are generally very different from country to country. For example, the primary reform goals for developed nations most likely focus on seeking equitable access to care, a high level of public satisfaction, and high quality of care while controlling costs. Meanwhile, countries with emerging economies may focus on delivering high-quality care at costs that are affordable for growing middle class populations. In contrast, a top priority in the healthcare agendas of developing countries would be to provide access to basic healthcare for as much of the population as possible within the constraints of limited funding.

The continuum of international health systems can be differentiated by the role of the government versus the market. For example, the health systems of developed nations, such as the United Kingdom and Switzerland, are predominantly government run--health institutions are mainly held and operated by the government. In contrast, administration and oversight of the health system in the United States (U.S.) is fundamentally rooted in the concepts of a market economy, although the government has provided health insurance coverage for the poor and the elderly since 1965. Japan and Germany are two examples of countries that fall between the two modes mentioned above--the government plays an important role to varying degrees.

China is the most populous country in the world, with over 1.3 billion citizens. It has an emerging economy, but is still a low-income country--ranking as the world's second largest economy, but 93^rd ^in gross domestic product (GDP) per capita. Uneven regional economic development across China has led to a widening gap between the rich and the poor. The 2007 Gini coefficient was 0.48--an increase from the 1981 Gini coefficient of 0.30, but higher than the U.S. value of 0.43 [2]. The current healthcare reform priorities of China lie in providing broad access to basic healthcare services for the poor, while also enhancing quality of care and keeping costs affordable for middle-income citizens. Healthcare reform in China is urgently needed, and existing gaps between the rich and the poor, as well as vast regional differences, suggest that authorities must adopt different financing structures and approaches to reform that are tailored to the needs of each region, rather than relying on a "one size fits all" approach. Advancing primary care in China is one strategy that could help reduce disparities in access to, affordability of, and quality of healthcare for all citizens. This article addresses the following three main issues: (1) conceptualizing primary care, (2) evidence of primary care effectiveness, and (3) promoting primary care to improve healthcare delivery.

## Conceptualizing Primary Care

### Defining primary care

In 1978, the World Health Organization (WHO) defined "primary care" during the International Conference on Primary Health Care in Alma-Ata as care that is (1) universally accessible to individuals and families in communities, (2) available at an affordable cost to communities and countries, and (3) the first level of contact for patients (or the first element of a continuing healthcare process) [3]. The U.S. Institute of Medicine put forth a similar definition in a 1978 report, and went on to specify the term "primary care practitioners" as inclusive of physicians, nurse practitioners, and physicians' assistants [4]. In fact, the primary care workforce should be far more expansive, and also include dentists, social workers, mental health specialists, public health practitioners, and community outreach workers.

### Primary care attributes

Dr. Barbara Starfield was the first researcher to define four cardinal attributes of primary care: (1) first contact, which emphasizes accessibility; (2) longitudinality, which describes continuity of care; (3) comprehensiveness, which describes the scope of care as providing health services for common health problems; and (4) coordination, which refers to the integration of care within the healthcare system [5]. Primary care plays the role of a gatekeeper in the healthcare system--patients are referred to specialists only when health problems are too unusual or complex. Primary care includes a wide range of services. In addition to providing adequate basic healthcare services, it also assumes the responsibilities of ensuring public health, and conducting chronic disease management, home care, mental healthcare, and other services.

### Distinction between "primary care" and "primary healthcare"

While primary care focuses on individuals, primary healthcare focuses on the population and communities. In other words, primary healthcare is primary care that is applied at the population level. Primary healthcare encompasses public health interventions, and requires the commitment of governments to develop population-oriented programs and services that target various determinants of health.

### Measurement of primary care

Given the mounting evidence that primary care is strongly associated with health outcomes, efforts to assess and assure the quality of primary care service delivery are important. The Johns Hopkins Primary Care Assessment Tools (PCAT), developed by the Johns Hopkins Primary Care Center, consist of a series of scales to evaluate primary care, and include consumer-client surveys, facility surveys, provider surveys, and health system survey [6]. The surveys were designed with questions that could be objectively measured such as the following question that can be answered dichotomously (with a yes or no response): "can you be seen by a practitioner from your usual source of care during the weekend?" Adult consumer surveys are used to evaluate accessibility of primary care, and commonly include questions on satisfaction with care. The related surveys in PCAT do not include such questions, because they are often influenced by consumer expectations of healthcare that can widely vary and are difficult to objectively assess. For more details about the assessment tools, please visit the following web site: http://www.jhsph.edu/pcpc/pca_tools.html.

## Evidence of Primary Care Effectiveness

### The relationship between primary care and health care expenditures

**Figure 1:**
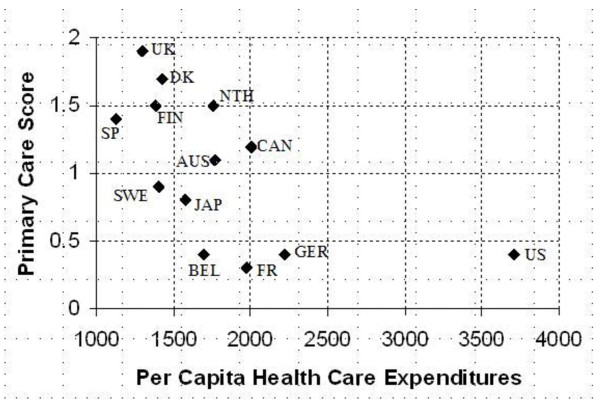
**Primary Care Score vs. Health Care Expenditures**.

One study conducted by researchers of the Johns Hopkins Primary Care Policy Center examined the "degree of concern" that several developed countries place on primary care, using a questionnaire to score primary care from 0 to 2 (0 represented poor performance of primary care and 2 represented good performance of primary care) [7] Results from this study showed that a country's degree of concern on primary care is inversely proportional to per capita health expenditures (Figure 1), and suggest that primary care can lead to savings in medical costs.

### The contribution of primary care systems to health outcomes

**Figure 2:**
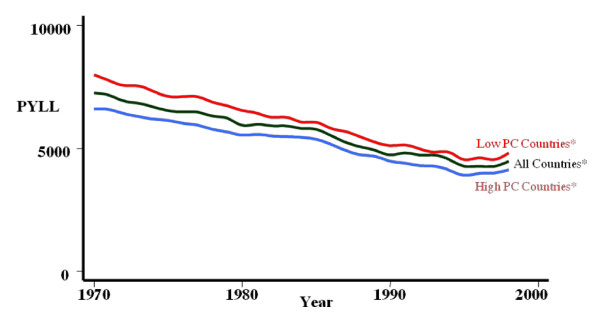
**The comparison of health outcomes between the high PC countries and the low PC countries (1970-2000)**.

A second study conducted by researchers of the Johns Hopkins Primary Care Policy Center set out to assess the association between primary care systems and health outcomes. The developed countries being studied were divided into two groups based on "high primary care" (High PC) and "low primary care" (Low PC). Potential years of life lost (PYLL) was used as a measure of health outcomes. This study found that although all countries had improved health outcomes in 2000, compared with 1970, the PYLL of High PC countries was lower than that for Low PC countries (Figure 2) [8]. These findings suggest that strong primary care systems and practices can play an important role in the improvement of health outcomes.

### The US-based studies

**Figure 3:**
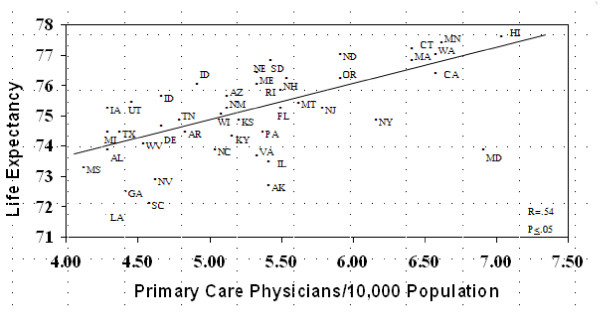
**The Relationship between Primary Care and Life Expectancy in 50 U.S. States**.

In order to study the relationship between primary care and health outcomes, researchers in this paper conducted a third study using data from the U.S. The U.S. degree of concern on primary care was measured using data on the number of primary care physicians/10,000 population, while data on life expectancy were used as a measure of health outcomes. Results showed that the number of primary care physicians/10,000 population was positively associated with life expectancy, which suggests that advancing primary care could be helpful in improving health outcomes (Figure 3) [9].

We also conducted analyses to control for potential confounding effects and to further examine the effects of primary care on health outcomes. Multiple regression coefficients of primary care physician-population ratios, income inequalities, and effects of smoking on health outcome indicators were weighted for the 50 states in the U.S. Results again showed that primary care may exert a health-enhancing role on most of the outcomes that were examined (Table 1) [10].

**Table 1:** Multiple Regression Coefficients of Primary Care Physician-Population Ratio, Income Inequality, and Smoking on Health Outcome indicators

	Total Mortality	Stroke	Neonatal Mortality	Post Neonatal Mortality	Life Expectancy
Primary care	- *	- *	-	- **	+ **
Income Inequality	+ **	+	+ *	+	- **
Smoking	+ **	+	+ *	-	- **
R2	0.65	0.19	0.28	0.21	0.54
Adjusted R2	0.63	0.14	0.23	0.15	0.51

Note: *P < 0.05, **P < 0.01

In sum, the above research findings demonstrate that the presence of primary care providers are significantly associated with improvements in health at both population and individual levels, and also suggest that primary care can reduce income and racial/ethnic disparities in health. Countries with healthcare systems that are specialist-oriented appear to be more costly and demonstrate much slower and smaller improvements on population and individual health.

### Promoting Primary Care to Improve Healthcare Delivery in China

There must be a shift in healthcare delivery within China, in light of changing population demographic trends (i.e., aging and longer life expectancies), as well as a paradigm shift from acute illness to chronic disease. Healthcare systems must focus on wellness rather than illness, on preventive care rather than acute care, on community well-being rather than merely individual health, on integrated delivery system rather than independent institutions and fragmented care, and on a continuum of services rather than service lapses or duplication of care. In 2009, the Chinese government adopted a health reform law that aimed to provide universal health care to the population over the course of a decade [11]. Reform efforts involved a massive expansion of health insurance to cover most Chinese citizens, as well as increased support for health care infrastructure throughout the country. While these efforts address some problems with the health care system, we believe that a healthcare system that follows a national health policy emphasizing primary care would go even further to address health challenges among populations. Recommendations for China's health reform are as follows.

### Build a balanced health workforce centered around primary care

A health workforce centered around primary care providers would move away from a specialist-dominant workforce and the subsequent pursuit of costly medical technology. Under this model, primary care providers would earn approximately the same income as medical specialists. In addition, financial and professional incentives could be made available to attract talented providers to the field of primary care, and encourage them to work in remote and rural communities where their services are most needed.

### Fundamentally overhaul the payment and financing structure

No healthcare system can be sustained by a reliance on drugs and the use of medical technology. As such, payment schedules must reflect the value of physicians' time and labour. Health insurance should provide incentives for patients to use primary and preventive care, rather than only catastrophic coverage. The government must provide coverage for the most vulnerable populations, including those who are low-income, the elderly, and children.

### Embrace the primary care team concept

A system that relies on physicians to provide primary care is neither sustainable nor effective. Instead, a primary care team concept should be embraced that consists of physicians, nurses, social workers, dentists, mental health specialists, community outreach workers, and volunteers. Primary care is not limited to clinical care; its comprehensive nature also encompasses counseling, social support, mental health, chronic care--all of which can be effectively and efficiently rendered by a well-balanced primary care team.

### Emphasize systematic and evidence-based research

Policy makers often seek "magic bullets" or quick fixes for a nation's problems, often at the expense of science. Research results are often distorted or incorrectly applied to justify a previously-held position. This practice is dangerous to the topics under study, as well as to the development of the research field. Policy makers must realize the importance of evidence-based research that is painstaking and independently conducted. Researchers must also be pragmatic and understand the policy implications of their work. In the case of China, well-designed demonstration projects must be conducted in different locales in order to generate research prototypes for different communities that can be put into practice.

### Learn from the international experience

When setting out to conduct healthcare reform, China should look to similar and successful reform efforts in other countries, which have accumulated a wealth of experience and lessons. These efforts could be studied and critically incorporated into China's healthcare reform. The vastness of China dictates that no single system could fit all regions; thus, national policy must be tailored to local resources and initiatives.

## Competing interests

The authors declare that they have no competing interests.

## Authors' contributions

LH and LS conceptualized the study, and wrote an early draft. SR and JT participated in the design of the study and helped with the analyses. All authors read and approved the final manuscript.
